# Agent swarms: Cooperation and coordination under stringent communications constraint

**DOI:** 10.1371/journal.pone.0311513

**Published:** 2024-12-11

**Authors:** Paul Kinsler, Sean Holman, Andrew Elliott, Cathryn N. Mitchell, R. Eddie Wilson

**Affiliations:** 1 Department of Electronic & Electrical Engineering University of Bath, Bath, United Kingdom; 2 Department of Mathematics, University of Manchester, Manchester, United Kingdom; 3 School of Mathematics and Statistics, University of Glasgow, Glasgow, United Kingdom; 4 Department of Engineering Mathematics, University of Bristol, Bristol, United Kingdom; Chunghwa Telecom Co. Ltd., TAIWAN

## Abstract

Here we consider the communications tactics appropriate for a group of agents that need to “swarm” together in a challenging communications environment. Swarms are increasingly important in a number of applications, including land, air, sea and space exploration, and their constituent agents could be satellites, drones, or other autonomous vehicles. A particularly difficult problem is to autonomously connect a swarm of agents together in a situation where stringent communication constraints are present, whether due to a need for stealth, restricted on-board power, external requirements to avoid certain broadcast directions, or equipment & hardware limitations. Here we present a novel, discrete, geometry-free model applicable to multi-agent swarm communications where a group of agents need to connect together and where the constraints on the communications dominate the algorithmic outcomes. No global knowledge of the agent locations is held and hence our framework proposes agent-centric performance metrics. We demonstrate our model using a set of candidate connectivity tactics and we show how simulated outcome distributions, risks and connectivity depend on the ratio of information gain to information loss. We also show that checking for excessive round-trip-times can be an effective minimal-information filter for determining which agents to no longer target with messages. The framework and algorithms that are presented here have wider application in testing efficient communication tactics across agent swarms in designated scenarios and testing the connectivity outcomes for future systems and missions.

## 1 Introduction

For more than a decade, the availability and range of applications for Unmanned Aerial Vehicles (UAVs) or “drones” has greatly increased. They now span areas such as logistics, agriculture, remote sensing, communications, and security [1–8], and in the future will become important for space exploration missions [9]. In particular, UAVs appear to be useful for tasks which are too dangerous, expensive, or inaccessible by crewed vehicles. Using a swarm also has the advantage of being robust against losses of individual elements.

Combining a group of drones into a “swarm” is a difficult engineering problem with potential to draw from a wide range of disciplines [10–13]. Further, investigations of such scenarios range from the relatively abstract [14–16]—as is our approach in this work—all the way through to a focus on specific applications such as search and rescue [11, 17, 18]. Indeed, swarm robotics and swarm engineering are emerging fields [13, 19–21] which cover automated decision making and control for large groups of agents using only local communication between nearby swarm members. There are special challenges inherent in the creation and real-time maintenance of non-centralised ad hoc networks [11, 22, 23] for groups of UAVs. Note that these are variously termed FANETs (Flying Ad Hoc Networks) [1] and UAANETs (UAV Ad Hoc Networks) [24]; these have been studied in recent years and various architectures and protocols have been proposed to handle communication and control of swarms [1, 3, 17, 18, 24–26]. However, most work does not consider network resilience to external attacks or the need to minimise risk of detection, although there has been research on autonomous swarms with covert leaders [27]. Additionally, the desire to control throughput has motivated some authors to propose network topologies and communications protocols aimed toward reducing the required communication overhead [25].

In this paper we focus on scenarios in which a swarm, consisting of multiple agents (which could be UAVs), must act in an extreme limit of minimal information sharing. This “cooperation under a communications constraint” is most easily represented as situations where communications must be restricted in order to maintain stealth, but can also be important in situations where we may wish to limit network throughput, such as with LoRaWAN [28, 29]. This is very different to typical scenarios, where information sharing and other communications are considered as “free”—i.e. they are not considered as an important factor in the communications algorithms. In these, each agent almost automatically has an excellent knowledge as to the state of the swarm, or at least some coordinating agent or supervisor has such information with which to efficiently direct swarm operations. It should be noted that this “cooperation under communications constraint” is a different problem to *control* under communications constraint [30, 31]; although e.g. [25] considers estimates on the maximum “throughput” allowed in drone networks as the size increases, and uses this to justify considering network protocols that minimise communication by utilising clustering approaches.

In the minimal information cases that we examine in this paper, agent to agent communications will, while accurate, be sparse. Therefore we must consider the cases where each agent is likely to have considerable uncertainty about many aspects of its surroundings, including the position of other agents, and understand that this information—and its reliability—will continually change with time. What this means in practice is that many of the typical treatments of swarm activities as given above (e.g. [1, 3–6, 10, 11, 19, 21]) become secondary problems to the fundamental issue [32]: i.e. how well does each agent know where the others are, so that it might send a message, and what tactics should it use to maximise the accuracy of its beliefs, whilst minimising its communications? A game theory [33, 34] problem arises here because an agent gains no new information by sending a message, but—in a “stealth” scenario—such transmissions only expose it to a risk of being detected. On the other hand, remaining silent could allow an agent to safely gather useful information from the transmissions of others. However, if *all* agents remain silent the agents cannot form a cooperating swarm as is the intent. The situation is therefore comparable to an inverse tragedy of the commons [35].

The main contribution of this paper is the conceptual framework and algorithms that demonstrate a new mathematical multi-agent information model. These provide an algorithmic capability that can be tailored to both continuum communications and discrete communications models. The discrete communications model is used in the design of a stochastic simulation code, which can test strategies to optimise communications whilst minimising risk.

We present our model and its concepts in Sec. 2. This is followed by a continuum communication implementation in Sec. 3, where an agent might act to modify its transmission priorities on the basis of the *rate* of information arrival from other agents. To provide measures of performance, we define agent and swarm metrics in Sec. 4. Then we describe our implementation of a discrete communications approach in Sec. 5, where we use a Monte-Carlo approach to understand the distribution of possible outcomes. In Sec. 6 we show and explain some results using this approach. After a discussion in Sec. 7, we conclude in Sec. 9.

## 2 Methods and model: Overview

In this section the model is introduced. In our framework, agents both cooperate and coordinate. Here cooperation refers to the necessity that all agents must cooperate by informing and updating the others about their locations. Without this, agents will end up being unable to target messages successfully. Next, coordination can only follow if the information for transmission targetting is well distributed enough that a fully connected swarm might be formed, i.e. a swarm where messages can be routed from one agent to another, possibly using multi-hop message paths (see Sec. 4.3). Without multi-hop messaging, any message could only be sent directly between individual agents. In such a direct-signalling model, not only would an increased transmission power be needed to reach the longer ranges, increasing both risk and energy expenditure, but any single blocked messaged path would prevent full swarm connectivity.

Therefore we consider a swarm of *N* agents that intend to cooperate and transmit information updates on their locations to each other. Our model is intended to mimic the behavior of a spatially distributed swarm of agents, but without requiring a detailed spatial model and all the additional complications that would entail. As such it contains only a minimal set of features and is primarily intended to facilitate an understanding of how the particular scenario of “cooperation under a communications constraint” might be handled. The model includes a representation of an agent’s current knowledge (or so-called beliefs, since the knowledge is limited in completeness and accuracy), the degradation of that accuracy as time passes, the environment’s effect on signalling efficiency, and agent information transmission rates. Thus:

***First***, each agent *a* has an information store about all other agents *j*, which we summarize using $\Phi_{j}^{a}$. If this store contains recent and reliable information, we would expect it to result in a high probability of a transmission being successfully received, but if the information is outdated or otherwise unreliable, the probability would instead be low. Thus these accuracies are represented as probabilities, using real numbers $\Phi_{j}^{a}\in \left[0,1\right]$; with 0 representing entirely inaccurate and misleading beliefs and 1 representing perfectly accurate beliefs. We assume here that any agent *a* is perfectly informed about its own state, and so set $\Phi_{a}^{a}=1$.
***Second***, we allow for the possibility that an agent’s beliefs about others slowly become out of date and degraded. We model this by assuming that all the $\Phi_{j}^{a}$ (iff *j* ≠ *a*) decay exponentially as determined by some loss parameter *γ*. However, we also assume there is a “find by chance” probability Φ_m_, so that this value is the “target” value of the decay. In most situations, we might expect $\Phi_{j}^{a}\geq \Phi_{\text{m}}$ to hold for any *a* and all *j*. Since if an agent *a* has *no* beliefs about some other agent *j*, it could guess where they were by chance, then $\Phi_{j}^{a}=\Phi_{\text{m}}$ would hold.
***Third***, there is an agent-to-agent transmission efficiency, which represents environmental constraints that might hinder communications between agents. This agent-to-agent transmission efficiency *L*_*ab*_ ∈ [0, 1] enables the representation of a wide range of networks, including networks based on spatial positions and signal models, as well as those with *L*_*ab*_ generated randomly according to some algorithm, e.g. abstract Erdos-Renyi (ER) networks [36]. However, at this early stage we do not specify how the efficiencies *L*_*ab*_ might have been generated, and can even allow *L*_*ab*_ ≠ *L*_*ba*_, i.e. the transmission efficiency from *a* to *b* may be different to the transmission efficiency from *b* to *a*. An important feature is that we do not assume any agent *a* has any information about either *L*_*ba*_ or *L*_*ab*_.
***Fourth***, since an agent might transmit information at different rates towards different targets, we also specify its set of transmission rates $\alpha_{i}^{a}$.

There is important distinction to make with regard to inter-agent communications, i.e. transmissions. Primarily, we say that an agent *a* can transmit an ***update***, i.e. information about its location, to other agents *j* (and hence improve their $\Phi_{a}^{j}$); optimising these is the main concern of our analysis. However, there is also a crucial secondary possibility, that of (also) transmitting a ***message*** to other agents, possibly for subsequent forwarding. In this paper, we will use “update” only to refer to transmissions aimed at improving the receiver’s information stores Φ, and “message” only to refer to this second function.

**Fig 1:**
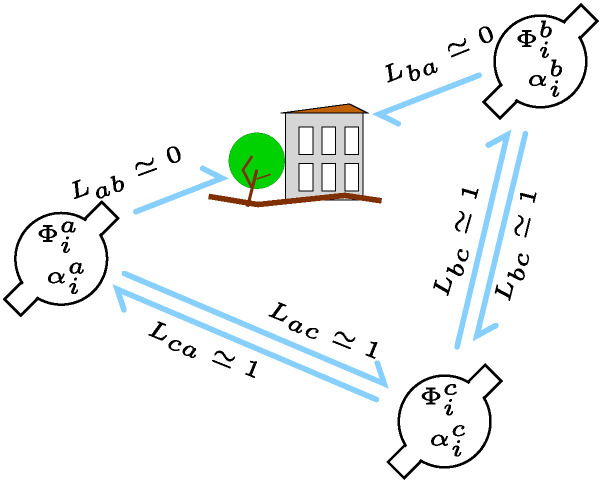
Diagram of a simple swarm with three agent-drones *a*, *b*, and *c*; where the link *a* ↔ *b* is blocked so that *L*_*ab*_ and *L*_*ba*_ are near zero, whereas the *a* ↔ *c* and *b* ↔ *c* links are free of obstruction. This means we would expect the values $\Phi_{b}^{a}$ and $\Phi_{a}^{b}$ to be small (since the beliefs, being poorly updated, will become ever more inaccurate), whereas the unobstructed messaging along links *a* ↔ *c* and *b* ↔ *c* should mean that it is possible to maintain $\Phi_{c}^{a}$, $\Phi_{a}^{c}$, $\Phi_{c}^{b}$, and $\Phi_{b}^{c}$ at values near 1.

These definitions mean that while the probability of successful information update (or message) transmission from an agent *a* to another *b* is straighforwardly given by the product of $\Phi_{b}^{a}$ and *L*_*ab*_, the actual rate of information arrival is $\alpha_{i}^{a}\Phi_{b}^{a}L_{ab}$. This model is broadly consistent with an implict assumption that transmissions are sent directionally and *need* to be aimed, being sent from *a* to *b* with an accuracy $\Phi_{b}^{a}$ across a link with efficiency *L*_*ab*_. Also, in this model, transmissions are only ever received by the intended recipient. A simple depiction with just three agents and one blocked (inefficient) link is given in Fig 1.

In this work, we do not set the environment property *L*_*ab*_ according to any model of signal transmission, or of a simulated environment, but instead we choose values in order to investigate specific cases. The two main cases are either a fully connectable swarm where all *L*_*ab*_ are set to the same near-unity value, or a partially connectable swarm where *L*_*ab*_ values are randomly selected as either zero or one.

### 2.1 Index convention: Subscripts and superscripts

To enable easier interpretation of the model parameters and values, we use an index convention where each indexing letter, and its positioning as a super- or sub-script, implies extra meaning. If we are referring to some specific agent we use one of {*a*, *b*, *c*}, where *a*, *b*, *c* ∈ {1, 2, …, *N*}; but if referring to a range of other agents will use one of {*i*, *j*, *k*}, where *i*, *j*, *k* = {1, 2, …, *N*}. Further, a superscript denotes that the quantity is a property of that superscripted agent, but for a subscript, there is no such implication. That is, the value $\Phi_{j}^{a}$ is a property of *a* for any *j*, but for none of those *j* (if *a* ≠ *j*) is it a property. Thus $\Phi_{b}^{a}$ is a number that is a property of agent *a*, and $\Phi_{i}^{a}$ is a collection of numbers that is a property of agent *a*. However, the collection of $\Phi_{j}^{i}$ (or even $\Phi_{a}^{i}$) is not ever completely known by any single agent. This is because *i* and *j* each encompasses many agents, so that $\Phi_{j}^{i}$ is not a property of any single agent in the swarm. Other characters used as sub- or superscripts will indicate not agents but special cases or particular values of e.g. Φ or *L*. We do *not* use any implied summation convention.

### 2.2 Abstractions are not knowledge

When using models of the type proposed here, it is important to note that an agent property (e.g. $\Phi_{i}^{a}$) is defined within the model as being attributable to an agent *a*, and may affect the outcomes of *a*’s actions. However, even though the model of agent *a* contains a collection of properties, this *does not* mean that the agent decision making can necessarily make use of each and every agent property. In particular, here we have that $\Phi_{i}^{a}$ is a representation or *abstraction* of agent *a*’s beliefs about the spatial location of agent *i*, thus telling the model how efficiently agent *a* can target that agent *i*. This is why the model will use it when calculating either what fraction of the information contained in the transmissions sent actually arrives, as in the continuum communications model; or alternatively whether or not a complete transmission is received, as in the discrete communications model.

Despite this, there is no reason why any *actual* agent *a* will be aware of and be able to use the value of $\Phi_{i}^{a}$ in decision making. For example, the model could specify that an agent *a* has an accuracy $\Phi_{b}^{a}=0.50$ when messaging *b*. However, the agent *a* might not be *aware* that that is the accuracy, so that it cannot use its value of 0.50 when decision making, e.g. by using it in a formula or algorithm. This is because an actual agent will instead only be cognisant of some specific “basket” of data—containing entries such as position estimates, likely errors, future plans for movement, and so on—which need not be reducible in an algorithmic way to the model’s substitute, i.e. the abstraction $\Phi_{i}^{a}$’s particular value. That is, any *actual* agent *a* might only be aware of a basket of specific details, but not how to synthesise the abstract $\Phi_{j}^{a}$ from those details. Indeed, when *we* use this model, we do not know this synthesising process either, nor anything about the basket contents.

**Fig 2:**
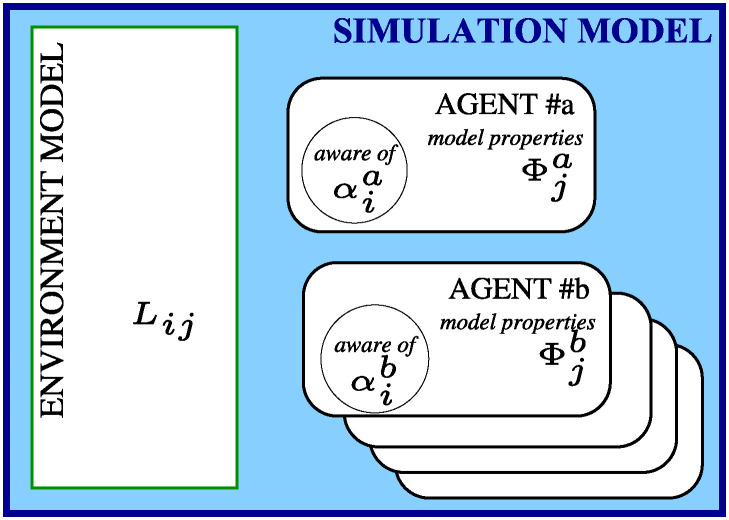
Schematic showing model (or simulation) parameters, i.e. environment (*L*_*ij*_) and agent properties ($\Phi_{a}^{b}$, $\alpha_{a}^{b}$), and indicating that an agent is only aware of some of the agent model’s properties (here, only $\alpha_{a}^{b}$). Depending on the interpretation intended by a specific model, the information loss *γ* might be in any one of these categories; or it might even be a combination of both environment and agent properties.

As discussed above, and as indicated in Fig 2, we have that $\Phi_{i}^{a}$ is a property of the agent *a* model, but that agent *a* is not aware of $\Phi_{j}^{a}$. In contrast, an agent should be aware of its choices or settings for transmission rates $\alpha_{i}^{a}$, so these would be usable in decision making. However, whether parameters such as the knowledge decay (*γ*) or the find-by-chance probability (Φ_m_) are agent properties that the agent is aware of, agent properties that are unknown, or even parameters entirely unrelated to the agent description, will depend on how we envisage the model of agent loss and information transmission. Nevertheless, since an agent *a* is unaware of the agent property $\Phi_{j}^{a}$, it seems reasonable to decide likewise that *γ* is also (at best) only an (unknown) agent property. However, if *γ* were (e.g.) dependent on the environment, it might not even be considered an agent property.

This distinction between the model’s abstractions and an agent’s actual awareness or beliefs—whatever they might be—means that to implement a generalisable communications tactic we must avoid reliance on our model’s abstractions, and instead use only quantities that an agent is aware of, can measure, or believes.

### 2.3 Terminology

In this work we will often refer to a “link”, meaning the potential for communication between two agents *a* and *b*. However, here “link” is just a short and convenient word for that potential, and it does not imply that such a communication is guaranteed to be easy—or even possible—in any particular case. When discussing links, we will also use three adjectives—efficent, accurate, reliable—to describe them; and in our context these three adjectives have specific and distinct meanings, which we will now define.

**Efficient**: for some link between two agents *a* and *b*, we say that there is an “efficient” link if *L*_*ab*_ is sufficiently large, i.e. if it exceeds some suitable threshold value, as discussed later—e.g. perhaps if *L*_*ab*_ > 0.75; conversely it is an “inefficient” link if it does not.
**Accurate**: when considering targetting, for some link between two agents *a* and *b*, we say that there is “accurate” targetting of transmissions from *a* to *b* if $\Phi_{b}^{a}$ is sufficiently large, e.g. if it exceeds some suitable threshold value; conversely it is “inaccurate” if it does not.
**Reliable**: for some link between two agents *a* and *b*, we say that there is a “reliable” link from *a* to *b* if the product $\Phi_{b}^{a}L_{ab}$ is sufficiently large, e.g. if it exceeds some suitable threshold value; conversely it is an “unreliable” link if it does not.

When communicating over these links, the agents will send transmissions, and depending on the model, this may deliver information either gradually and incrementally, as in our continuum communications model; or in packets, as in our discrete communications model. The primary purpose of these transmissions is that they enable accurate targetting of replies back to the sending agent, but in our discrete model they also contain timing information as to when the last transmission on that link was received.

However, this terminology is only intended to make general discussion clearer by specifying preferred adjectives, rather than as any unique mathematical specification; and for descriptive purposes the exact thresholds are not of primary importance.

## 3 Model: Continuum communications

In this section we describe a continuum model which represents a system in which all agents are simultaneously feeding trickles of update information to all other agents, with no randomness or contingency. This provides us with a good starting point with which to introduce parameters and concepts in a simple and direct way. Later in this paper we will use a more plausible stochastic model, based on discrete communications (see Section 5).

First, we assume that each agent *b* transmits to each other agent *a*, sending information updates about itself (only) according to an information rate $\alpha_{a}^{b}$, and with a targetting accuracy dependent on its imperfect $\Phi_{a}^{b}$. Further, the information stream sent will be attenuated in transit by the link efficiency *L*_*ba*_.

As a result *a* will only be able to improve its $\Phi_{b}^{a}$ at a maximum rate $q_{b}^{a}=\alpha_{a}^{b}L_{ab}\Phi_{a}^{b}$. Further, when agent *a* receives some information, it will only find the currently unknown part of that information useful. Thus only a fraction $1-\Phi_{b}^{a}$ of that arriving from *b* will add to the existing total $\Phi_{b}^{a}$.

Here we assume that this receive rate $q_{b}^{a}$ can be measured therefore the receiving agent will be aware of it, but that the agent cannot measure its constituent contributions $\alpha_{a}^{b}$, *L*_*ab*_, and $\Phi_{a}^{b}$ individually. However, we do allow that the receiving agent might still be able to make plausible inferences about those unknown contributions by making some assumptions.

### 3.1 Dynamics

Based on the description and parameters given above, we can now write a rate equation for the behaviour of each $\Phi_{b}^{a}$, for *a* ≠ *b*, which is

$$\frac{d}{dt}\Phi_{b}^{a}=-\gamma (\Phi_{b}^{a}-\Phi_{\text{m}})+\alpha_{a}^{b}L_{ba}\Phi_{a}^{b}(1-\Phi_{b}^{a}).$$ (1)

In what follows we will typically assume that the *γ*, *L*_*ba*_, $\alpha_{a}^{b}$, and Φ_m_ values are fixed parameters, and only the $\Phi_{b}^{a}$ are time-dependent.

This framework also allows more general scenarios. For example, we could additionally permit an agent *b*’s beliefs about its own position to not be perfectly accurate, i.e. allow $\Phi_{b}^{b}<1$. In such a case, the second term on the right hand side of (1) should also be multiplied by $\Phi_{b}^{b}$; since it is passing on imperfect information. However, we might also need an additional or modified rate equation to determine how each of $\Phi_{i}^{i}$ might behave; or reinterpret $\alpha_{a}^{a}$ or *L*_*aa*_ as providing a supply of new self-location information.

### 3.2 Steady state

Since there are no complicated interdependencies, it is straightforward to get a steady-state solution by setting $\left(d/dt\right)\Phi_{b}^{a}=0$. This gives

$$\begin{array}{c} \gamma (\Phi_{b}^{a}-\Phi_{\text{m}})=\alpha_{a}^{b}L_{ab}\Phi_{a}^{b}(1-\Phi_{b}^{a}) \end{array}$$ (2)

$$\begin{array}{c} \gamma \Phi_{b}^{a}-\gamma \Phi_{\text{m}}=\alpha_{a}^{b}L_{ba}\Phi_{a}^{b}-\alpha_{a}^{b}L_{ba}\Phi_{a}^{b}\Phi_{b}^{a} \end{array}$$ (3)

$$\begin{array}{c} (\gamma +\alpha_{a}^{b}L_{ba}\Phi_{a}^{b})\Phi_{b}^{a}=\gamma \Phi_{\text{m}}+\alpha_{a}^{b}L_{ba}\Phi_{a}^{b} \end{array}$$ (4)

$$\begin{array}{c} \Phi_{b}^{a}=\frac{\gamma \Phi_{\text{m}}+\alpha_{a}^{b}L_{ba}\Phi_{a}^{b}}{\gamma +\alpha_{a}^{b}L_{ba}\Phi_{a}^{b}}. \end{array}$$ (5)

Here we see that—as expected—if losses are small then each agent might achieve nearly perfectly accurate beliefs. Conversely, if losses are large, then in this idealised steady state each agent is only left with the “find by chance” value Φ_m_. The threshold between these two extremes is located in the regime where the effective information transmission rate $\alpha_{a}^{b}L_{ba}\Phi_{a}^{b}$ becomes comparable to the information loss rate *γ*Φ_m_.

**Fig 3:**
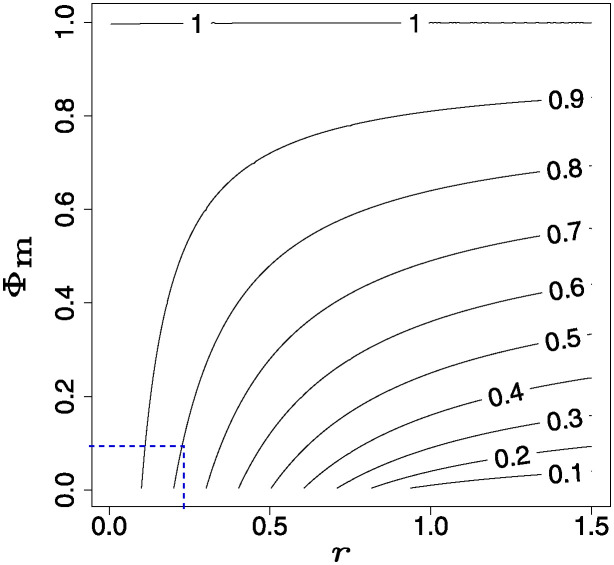
Contour plot of the steady-state targeting accuracy $\Phi_{b}^{a}$ values from (6), as dependent on the find-by-chance probability Φ_*m*_ (which is usually set according to the scenario) and the effective accuracy loss rate $r=\gamma /\alpha_{a}^{b}L_{ba}$. Note that the typical range of interest will be for small values of Φ_*m*_, and small *r*; i.e. that at the lower left part of the figure. As an example, if Φ_m_ = 0.10 then if we want to achieve a minimum 80% accuracy then we need to keep the effective loss rate *r* below 0.25 (as indicated by the dotted line); for 90% we would need *r* < 0.10. If the information loss parameter *γ* were fixed, then to improve accuracies $\Phi_{b}^{a}$ we would need to increase the transmission rates $\alpha_{b}^{a}$.

By using this *b* → *a* expression (5) in concert with its *a* → *b* counterpart, we can get a quadratic expression for $\Phi_{b}^{a}$ that tells us the informational effect of any single agent-to-agent link as we show in S1 Appendix. If we then assume symmetric parameters, i.e. with $r=\gamma /\alpha_{a}^{b}L_{ba}=\gamma /\alpha_{b}^{a}L_{ab}$, then we find that

$$[\Phi_{b}^{a}{]}^{2}+[r-1]\Phi_{b}^{a}-r\Phi_{\text{m}}=0,$$ (6)

which can be solved, with the valid (i.e. positive valued) solution being plotted on Fig 3. We see that in any scenario with a fixed minimum find chance Φ_m_, the performance (i.e. here the steady-state value of $\Phi_{b}^{a}$) will degrade if the information environment becomes more challenging (i.e. as loss *γ* increases), but with no sharp threshold behaviour.

### 3.3 Communication tactics: Payoffs and penalties

If we make a simple assumption that the risk or cost to an agent is simply proportional to the message volume it sends, any agent *a* will wish to minimise its totalled $\sum_{j}\alpha_{j}^{a}$ rates of data transmission. Clearly, therefore, it might want to set $\alpha_{b}^{a}=0$ if it can reliably infer that *L*_*ab*_ is already small, since this is an inefficient link and probably not worth maintaining. This optimization would be especially valuable if (e.g.) *a* might instead communicate with *b* via efficient links through some intermediate agent *c*. However, a low rate of incoming information $q_{b}^{a}$ could be due to any of three factors: (i) a low link efficiency *L*_*ba*_, (ii) a sending agent with inaccurate beliefs $\Phi_{a}^{b}$, or (iii) a sending agent which has chosen a low transmission rate $\alpha_{a}^{b}$.

Of course, *if* each agent *a* has an estimate of Φ_m_ and *γ*, and is assumed to actually be aware of the values of its set of $\Phi_{j}^{a}$, it could fix an $\alpha_{j}^{a}$, wait for steady state, assume symmetric behaviour, and then attempt to estimate the *L*_*ja*_ for each *j*. Given this, it could customise its $\alpha_{b}^{a}$ accordingly, although the assumption of symmetry—i.e. that the other agent(s) will be doing exactly the same thing, and at the same time—is a very aggressive one.

For example, for an ER network [36] in the limit where there is a large connected component, i.e. where the probability of an (*L* = 1) link between any two agents is *p* = log(*N*)/*N*, a 1 − *p* proportion of links might be pruned by such a process. Then, assuming that all non-zero (and non-zeroed) transmission rates $\alpha_{j}^{i}$ have some constant value $\alpha_{c}$, we see that the swarm risk rate likewise drops, i.e. from proportional to *α*_c_*N*(*N* − 1)^2^ to proportional to *α*_c_*N* log(*N*). However, coordinating all the agents—and remember they may all have very different and possibly changing beliefs—so that they all prune the available links down to compatible subsets will be a tricky problem, especially in this limited-communication regime.

## 4 Model: Agent and swarm metrics

Before moving to the discrete communications model that is used for the main results of this paper, it is useful to consider some agent and swarm metrics that can be used to judge how an agent or swarm instance is performing.

As set out in Sec. 2, we assume that inter-agent transmissions do not include agents forwarding any information updates from others: i.e. an update sent from *a* to *b* will not also contain information about a third agent *c*. This is a restrictive assumption, but one which greatly simplifies the model (cf. with the approach in [37]). It enables us to set some benchmarks for agent behaviour without introducing the considerable complications of how an agent might manage—and make inferences from—a diverse array of partial, uncertain, and variously out-of-date information about (e.g.) the locations of the other agents. Nevertheless, this restriction does not prohibit us from imagining the forwarding of (non-update) messages across the resulting network of communicating agents, and considering whether or not all agents might be connnected indirectly to each other.

### 4.1 Messaging rates

It is useful to assume that our agents have some maximum total transmission rate *A* that they are capable of; this also helps us set timescales on which the dynamics occurs, as well as assist conversion into the discrete model treated later. Thus we want to normalise $\alpha_{j}^{a}$ so that the *total* information transmission rate conforms to

$$\bar{A}\geq A^{a}=\sum_{j}\alpha_{j}^{a}.$$ (7)

We see here that an agent is not required to transmit at the maximum rate $\bar{A}$, and it can instead transmit at some reduced rate *A*^*a*^.

If one imagined that an agent *a* could infer a value for *L*_*ab*_, it could set $\alpha_{b}^{a}$ to zero if it believed *L*_*ab*_ too small to be worth trying to overcome; or increase $\alpha_{b}^{a}$ if it believed *L*_*ab*_ large enough to support a useful information flow.

### 4.2 Performance

A simple measure of how well informed an agent *a* is might be constructed by simply summing some “performance” function of the belief accuracies $\Phi_{j}^{a}$ and then applying some appropriate normalisation. The intent here is that the closer the performance measures suggested below are to unity, the more likely it is that the agents have sufficient good information with which to communicate effectively.

A simple link performance function of $\Phi_{j}^{a}$ might be ${\left(\Phi_{j}^{a}\right)}^{\beta }$, perhaps with just *β* = 1, but where choosing *β* > 1 will de-emphasise inaccurate beliefs in the measure. In this case, where the performance measure includes contributions from all $\Phi_{j}^{a}$ values, the normalisation should simply be 1/*N*. Thus the agent performance measure is

$$\begin{array}{c} {\bar{\Phi }}^{a}\left(\beta \right)=\frac{1}{N}\sum_{j}(\Phi_{j}^{a}{)}^{\beta }, \end{array}$$ (8)

and by extension the swarm performance measure is

$$\begin{array}{c} \hat{\Phi }\left(\beta \right)=\frac{1}{N}\sum_{i}{\bar{\Phi }}^{i}\;=\frac{1}{N^{2}}\sum_{i}\sum_{j}(\Phi_{j}^{i}{)}^{\beta }. \end{array}$$ (9)

However, this doesn’t work well for scenarios where each agent *a* doesn’t necessarily need to be aware of *every* other agent *j*, just a subset with hopefully good link efficiency *L*_*aj*_, that enables reliable connectivity over a sufficiently small number of hops. In such a case we might choose a suitable threshold value Φ_PT_ for the accuracy, and only include the “good” contributions, i.e. those where $\Phi_{j}^{i}>\Phi_{\text{PT}}$. That is, using the Heaviside step function *H*(*x*), we set the performance measure for an agent *a* to be based on

$$\begin{array}{c} h_{j}^{a}\left(\Phi_{j}^{a};\Phi_{\text{PT}}\right)=\Phi_{j}^{a}\;H\left(\Phi_{j}^{a}-\Phi_{\text{PT}}\right). \end{array}$$ (10)

and the normalisation ${\bar{N}}^{a}$ as the *maximum* of two possible values:

(i) a sum over accurate links, i.e.
  $$\begin{array}{c} {\bar{N}}^{a}\left(\Phi_{\text{PT}}\right)=\sum_{j}H\left(\Phi_{j}^{a}-\Phi_{\text{PT}}\right), \end{array}$$ (11)
  or
(ii) the ER average number of links per node when in the large connected component limit, i.e.
  $$\begin{array}{c} {\tilde{N}}^{a}=\text{log}\left(N\right). \end{array}$$ (12)

This “maximum of” is used as a pragmatic way to counteract misleading cases where an agent has (or agents have) too few beliefs that are sufficiently accurate to suggest any swarm connectivity, but where those that are accurate are nevertheless well above threshold, and so would otherwise return a misleadingly high performance measure.

Thus this thresholded agent performance measure ${\bar{\Phi }}_{\text{T}}^{a}$ is

$$\begin{array}{c} {\bar{\Phi }}_{\text{T}}^{a}=({\bar{N}}^{a}{)}^{-1}\sum_{j}\Phi_{j}^{a}\;H\left(\Phi_{j}^{a}-\Phi_{\text{PT}}\right), \end{array}$$ (13)

and by extension the corresponding swarm performance measure is

$$\begin{array}{c} {\hat{\Phi }}_{\text{T}}=\frac{1}{N}\sum_{i}{\bar{\Phi }}_{T}^{i}\;=\frac{1}{N}\sum_{i}({\bar{N}}^{i}{)}^{-1}\sum_{j}\Phi_{j}^{a}H\left(\Phi_{j}^{a}-\Phi_{\text{PT}}\right). \end{array}$$ (14)

**Fig 4:**
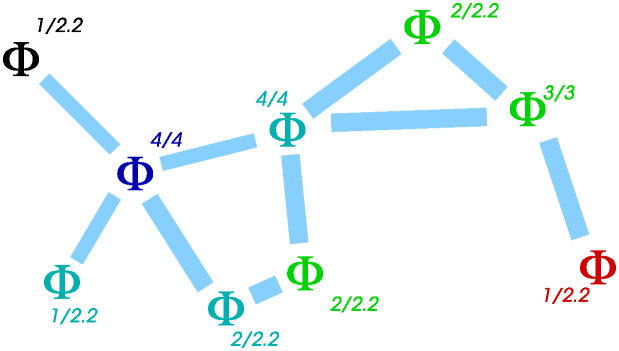
Example calculation of the ${\hat{\Phi }}_{\text{T}}$ performance metric. For this 9-agent swarm, with accurate links indicated with thick light blue lines, the “just connected” ER expected number of links per agent is log(9) ≃ 2.2; thus when working out the link-count normalisation we use this value even if the actual number of accurate links is 0, 1, or 2. Summing the contribution from each agent we get (1 + 1 + 2 + 2 + 2 + 1)/2.2 + (1 + 1 + 1) = 7.1, for a swarm performance measure of ${\hat{\Phi }}_{\text{T}}≃7.1/9=0.79$.

An example calculation is indicated on Fig 4.

Note that for any performance measure, whilst an agent *a* might calculate (or estimate) its own performance (e.g. ${\bar{\Phi }}_{\text{T}}^{a}$, as above), it cannot calculate the corresponding swarm performance (e.g. ${\hat{\Phi }}_{\text{T}}$).

As an aside, for any single set of link efficiencies {*L*_*ij*_}, we could compute a customised performance scheme that uses information about what actual links are good, as opposed to the approaches introduced above where we attempted a reasonable and *general* normalisation. However, since an agent is never aware of the true values of {*L*_*ij*_}, such a custom performance measure is not calculable by an agent attempting to determine whether it has sufficient good information about a sufficient number of nearby others.

### 4.3 Connectedness

**Fig 5:**
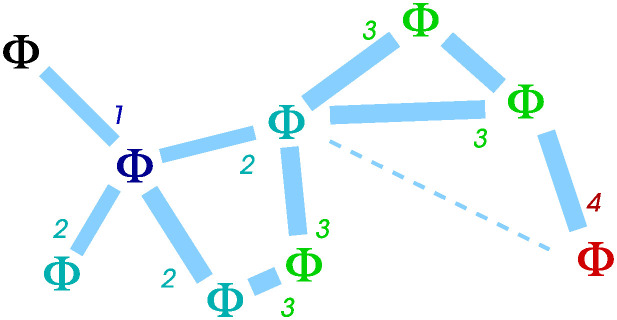
Counting the number of hops needed to pass a message from one agent to another. Here accurate links are indicated by the thick light blue lines, and these links are also assumed to be able to pass messages efficiently. In the situation shown here, although most agents are easily within three hops of one another, the two on the left and right extremes are four hops away from each other. Thus this swarm does not satisfy the CCS criteria, but it would if the dashed link were *also* accurately known. However, since it is the cumulative probability that counts for connectivity, many hop paths taken over imperfectly accurate links will in combination be less accurate (being the product of each hop accuracy), and may fall below the CCS probability-threshold criteria for being connected.

A “completely connected swarm” (CCS) is formed if—on the basis of $\Phi_{j}^{i}$ values alone—it seems that one might reliably send a message from any one agent to any other agent, optionally routing it via intermediate agents. Here, this determination is also subject to there being (a) no more than three such hops, and (b) that the probability of the message successfully traversing the whole path is above some suitably chosen probability threshold Φ_PT_; as indicated on Fig 5. Here we typically choose Φ_PT_ = 0.75 and three hops, as convenient criteria that provide representative results, and making CCS connectivity achievable, but not always guaranteed. This is a different measure than the large connected component of standard network theory, but is chosen to mimic plausible limitations on message passing across the swarm. That is, this choice of Φ_PT_ = 0.75 is chosen so as to cover scenarios where some message loss is present, but where messages are not routinely lost. Increasing the value of Φ_PT_ demands more reliable messaging, and makes it harder to achieve the CCS criteria; decreasing it makes it easier; but in general the results are not particularly sensitive to any specific choice.

This determination of connectedness does make the artificial assumption that the complete set of accuracies $\Phi_{j}^{i}$ could somehow be collected together, to enable a matrix of agent-to-agent message success probabilities. Thus, whilst a useful metric for judging the success of a simulation or some chosen messaging tactics, it is not something that any one agent can calculate.

Indeed, in this simple model here we have that each agent *a* is only aware of its own star-network; it has no way of inferring anything about connected components that involve hopping along multiple links. Any non-star “connected component” is not something an agent can ever be aware of, and therefore is not something an agent can use to make decisions. This is why we introduced the agent-centric performance measures ${\bar{\Phi }}^{i}$ above, since these tell us whether each agent is likely to have enough accurately linked neighbours in its star, so that a CCS (or even a large connected component) might exist.

Further, we *do not* include the effect of the link efficiencies *L*_*ij*_, because agents are always unaware of link efficiency values, and here there is also no attempt to estimate them. Clearly, this lack might be problematic, but if the *L*_*ij*_ all have values either near 1 or 0, the $\Phi_{j}^{i}$ should also tend to these values, so the discrepancy should be manageable.

### 4.4 Risk

As discussed above, here we consider the case where a swarm is attempting to remain undetected by some listening adversary; although other types of communications constraint might also be considered. Such other risk types would need their own risk models, which might be similar (such as avoiding signals interference) or rather different (avoiding running out of power). The rate ${\bar{R}}^{a}$ that the risk of detection accumulates to any agent *a* in this model is most simply assumed to be in proportion to a sum over its transmission rates $\alpha_{j}^{a}$. I.e., we have

$$\begin{array}{c} {\bar{R}}^{a}=\xi \sum_{j}\alpha_{j}^{a}, \end{array}$$ (15)

where *ξ* is a proportionality constant that converts a transmission rate into the rate of detection by some adversary. As a result, we can clearly see that the maximum transmission rate $\bar{A}$ set previously then also sets a maximum risk accumulation rate (“risk rate”) of $\xi \bar{A}$. However, an agent is not required to transmit at this maximum rate, so the actual risk rate can be smaller.

For all agents (i.e. the swarm), we then have the total

$$\begin{array}{c} \hat{R}=\xi \sum_{i}{\bar{R}}^{i}\;=\xi \sum_{i}\sum_{j}\alpha_{j}^{i}. \end{array}$$ (16)

Note that for any risk measure, whilst an agent *a* might calculate its own risk rate ${\bar{R}}^{a}$, it cannot calculate the swarm risk rate $\hat{R}$. Further, these risk rates are not the same as detection probabilities except in the limit where they are small, i.e. when ≪ 1. Instead, for a risk rate *R*′ that is present over an interval *τ* we can calculate the detection probability to be

$$\begin{array}{c} p=1-\text{exp}(-R^{\prime }\tau ). \end{array}$$ (17)

## 5 Model: Discrete communications

In this section the discrete model is introduced. Although many of the parameters in this model are identical to the continuum model above, our discrete communication model uses stochastic transmission and reception processes. This communications model is one which more closely matches a realistic situation of discrete information packets—whether of updates or of messages—being sent one after another to variously selected target agents. To simulate this system we wrote and used a bespoke Fortran code designed around this model and its planned extensions.

Here we assume that time passes in discrete steps (or “ticks” of duration *τ* each), and that in each tick each agent could at most transmit one packet aimed at any one other agent. For example, a trivial comunications tactic might be to select the target agent *b* at random from the list of other agents. This discrete communications model is very different to the continuum model which sends a continuous trickle of information to multiple other agents simultaneously.

Simulation parameters are set so that each agent sends a maximum of one update packet per tick, but over any period of *K* ticks, we set the goal of sending *N* updates. This goal of sending at a rate A* = *N*/*τK* is the counterpart of the “$\bar{A}$” parameter from the continuum model. Note that we need *K* > *N* to ensure that the maximum allowed transmission rate can be maintained, although a greater margin is preferable so that we can neglect the possibility of congestion effects. The transmission rate is reduced from its 1-per-tick maximum by applying conditions which need to be met before anything is sent, as we describe later in Sec. 5.2.

For reception, the link efficiency *L*_*ab*_ and the sender’s available information $\Phi_{b}^{a}$ can be used as a probabilistic filter to determine whether an update was received. If luck is on the receiver’s side, the packet arrives successfully and it gets all the information; if not, then it gets none. The outcome is binary—an update packet either arrives successfully and it communicates all of the information; or it fails.

### 5.1 Updates (dynamics)

The resulting information update equations for a tick of length *τ* can now be separated into two stages. For the loss update we alter each agent *a*’s information vector $\Phi_{j}^{a}$ according to

$$\begin{array}{c} \Phi_{j}^{a}\leftarrow \Phi_{\text{m}}+\text{exp}(-\gamma \tau )(\Phi_{j}^{a}-\Phi_{\text{m}}), \end{array}$$ (18)

and for the communications stage we update according to

$$\begin{array}{c} \Phi_{j}^{a}\leftarrow \Phi_{j}^{a}+W_{a}^{j}.(1-\Phi_{j}^{a}), \end{array}$$ (19)

where in any one tick, the $W_{a}^{j}$ is a yes-or-no (i.e. 1 or 0) list of all successful receptions at *a* of a (possible) transmission from all *j*. It is useful to split this $W_{a}^{j}$ into two parts, with $W_{a}^{j}=w_{a}^{j}b_{a}^{j}$, where $w_{a}^{j}$ and $b_{a}^{j}$ are defined next.

The first part ($w_{a}^{j}$) contains a “1” only if any agent *j* sends an update packet to target *a*. Note that for any sending agent *b*, only the entry for the update’s intended target *a* in $w_{i}^{b}$ is non-zero (i.e. $w_{i}^{b}=0$, except for *i* = *a*, since $w_{a}^{b}=1$). *Which* agent is chosen as a target in any given tick is now dependent on the chosen communications tactic (see below in Sec. 5.2) and the (average) probability with which an agent *a* is chosen as a target by *j* is—for small probabilities—related to the quantity $\tau \alpha_{a}^{j}$ from the continuum model.

The second part ($b_{a}^{j}$) is the reception filter, which will be one (i.e. unity) if the transmission is successfully received, and zero otherwise; i.e. if some random number $\eta_{a}^{j}$ chosen from a uniform distribution on [0, 1] is such that $\eta_{a}^{j}<L_{ja}\Phi_{a}^{j}$.

This means that in any given tick, $W_{i}^{j}=w_{i}^{j}b_{i}^{j}$ will be mostly zeroes, and have *at most* *N* entries that are one, and then only all *N* if each agent-sent transmission is lucky enough to successfully pass the reception filter. So here we see that unlike the continuum model’s gradual handling of information arrival and accumulation, in each tick here only some information elements are updated, and if they are updated, they are updated to become perfectly accurate beliefs, i.e. if *a* receives from *b*, then it sets $\Phi_{b}^{a}=1$. A summary of the operation of the simulation program is shown as Algorithm 1.

Regarding the normalisation, performance, and risk considerations present for the continuum model, here we have that:

1. The normalisation needed in the discrete model is more complicated than in the continuum model, where we could just set a maximum rate $\bar{A}$, i.e. just $\bar{A}\tau$ messages per tick. In the discrete model there is an absolute maximum for each agent of at most one packet per tick, but as described above we usually want to send fewer than this, and so set a goal of (at most) A*, i.e. *N* packets per time *τK*. Thus the relevant comparison is between the continuum model’s $\bar{A}$ and the discrete model’s goal of A*.
2. The performance measure(s) defined for our continuum model can be reused here, since they only depend on $\Phi_{j}^{i}$. However, since we now need to run large ensembles of instances of this model, we are not restricted to only considering only one outcome, or an averaged behaviour, of the $\Phi_{j}^{i}$ and values derived from it. We can also consider their distributions as accumulated over both time intervals and the many different instances.
3. The risk measure(s) defined for our continuum model can be reused here, but here they reduce to a simple packet counting. To go beyond this requires both a signalling model and an adversary model to be specified (e.g. see [32]).

**Algorithm 1**: The discrete communications simulation

1: initialise

2: **loop** over all times *t*:

3:  **loop** over agents *a* to apply *information decay*

4:   update all $\Phi_{j}^{a}$ using Eq (18)

5:  **end loop**

6:  **loop** over agents *a* to decide *transmissions*:

7:   use Tactic to select target other agent *i*

8:   set $w_{i}^{a}=1$ accordingly

9:  **end loop**

10:  **loop** over agents *b*; to check for *receptions*:

11:   **loop** over agents j; that could have sent to *b*:

12:    **if** $w_{b}^{j}=1$ **then** … there is *something* to receive

13:     set $B_{b}^{j}=(rnd<L_{jb}\Phi_{b}^{j})$

14:     **if** $B_{b}^{j}$ **then** … it was *actually* received

15:      apply update to $\Phi_{j}^{b}$ using Eq (19), i.e. set to 1

16:     **end if**

17:    **end if**

18:   **end loop**

19:  **end loop**

20:  save simulation state

21: **end loop**

### 5.2 Communication tactics

To understand the behaviour of our model we now consider a number of different communication tactics suitable for the discrete communications approach. Since we can no longer send to all other agents at once, but can only transmit to one at a time, how an agent chooses its target is important. Further, this discrete approach has the advantage that it is easily adapted to applications where individual agents are targeted along specific directions. This immediately suggests a simple tactic of targetting each agent sequentially, i.e. one-by-one in some fixed order. Of course other simple schemes are possible, most notably just choosing targets at random. More complicated tactics could involve choosing targets based on what updates were received and their timings. Any agent *a*, however, will need to consider the balance between the average number of updates sent to other agents *i* per tick (the counterpart of $\tau \alpha_{i}^{a}$ from the continuum model) and the belief decay rate (*γ*), and its effect on the performance of the other agents.

In the continuum case, each agent is aware of its transmission rates $\alpha_{i}^{a}$ and the receive rates $q_{j}^{a}$. Here, in analogy, agent *a* is aware of (i) $T_{j}^{a}$, how many ticks ago they last sent an update to the agent *j*, and (ii) $R_{j}^{a}$, how many ticks ago they last received an update from the agent *j*. Further, each transmitted update contains the sender’s matching receive-from time, so that a transmission there-and-back “round-trip” $O_{j}^{a}$ time can be calculated.

Although there is a large range of possible communications tactics, here we will restrict ourselves to a short list, three of which are closely related. We aim to address more sophisticated tactics in future work. The communications tactics we consider ensure that the updates of a sending agent are directed

(i) *Sequence*: towards each other agent in a fixed and repeating sequence;
(ii) *Random*: towards another agent chosen at random;
(iii) *Timer*: towards another agent chosen at random, as long as a time threshold has been exceeded;
(iv) *Filtered*: towards another agent chosen at random, as long as a time threshold has been exceeded; but not if that target agent has not reported sufficiently recent contact from the sender;
(v) *Filtered+* and *Filtered++*: as per Filtered; but there is also a probability of an extra update sent towards any other agent chosen at random, regardless of time delays or lack of contact. Filtered+ incorporates a 25% chance of considering such an extra update, whilst Filtered++ has 50%. Thus if there are ten agents in the swarm, these tactics choose randomly from ten possible target options; i.e. from the nine other agents plus the possible extra update.

The first three tactics here—i.e. (i), (ii), (iii)—do not attempt to select or deselect targets except as a rate-management technique; even inefficient or unreliable links will continue to be used. Although this does make them robust to unexpected changes in link efficiency, it also means they will waste updates and increase risk unnecessarily by transmitting over inefficient links. Nevertheless, they provide a set of baseline cases which we should be able to outperform.

The Filtered tactic (iv) proposed here adapts to the environment by excluding poor links. Its key feature is that it does this *without* requiring inferences about $\Phi_{i}^{a}$ or *L*_*ai*_ values. Only timing data is needed, and for a proposed update from sending agent *a* to target agent *b*, the only non-trivial requirement is that the last update from *b* received by *a* included the time $R_{a}^{b}$. This tactic, and its derivatives, are the only ones considered here that will on average send fewer updates than the goal rate A*, as excluded messaging possibilities are not redirected elsewhere, but dropped. However, once links have been deemed bad and dropped, they will never be recovered by this Filtered tactic. This is why we also introduce the Filtered+ and Filtered++ tactics (v) which—as we will see—delay this process.

## 6 Results

We now evaluate the performance of our five chosen communications tactics using the discrete communications approach in a static environment. We do not model the continuum version, since its “continually transmit to everyone, always” approach is unlikely to be of much practical use; although it did serve a useful function by introducing key concepts.

Here we will consider a swarm of *N* = 20 agents and contrast the cases of an in-principle fully-connectable swarm (i.e. where all *L*_*ij*_ ∼ 1) and a partially-connectable swarm where *L*_*ij*_ is a mix of zeroes and ones. The “hit by chance” minimum transmission targetting probability Φ_m_ = 0.10, which, if imagining a set of spatially distributed agents, is compatible with a *directed* transmissions covering an angle of 36°. However, in this model these transmissions are only ever received by their intended recipient, or they are not received at all. Updates are never received accidentally by some other (non-targetted) agent, which might happen in a true spatial description [32, 37].

Each simulation run starts with randomly chosen transmitted-to and received-from communications timings, thus setting up a plausible initial state. Nevertheless, since this is not guaranteed to be a good match to a typical quasi-steady state, we start by propagating for over 10 thousand (10k) ticks so that the distribution of agent properties should no longer be dependent on the initial conditions, and the evolution can be reasonably assumed to be ergodic. Then the next ∼ 20k ticks are averaged over in lieu of averaging over multiple independent simulations. In the following subsections we test all five of the tactics outlined previously in Section 5.2. the subsection on discrete communications tactics.

### 6.1 Fully connectable swarm

In a fully connectable swarm, every agent has a good chance of transmitting successfully to each other agent, i.e. *L*_*ij*_ ∼ 1. This makes it easy to successfully achieve a large, all-agent connected component, but there will likewise be a large number of good—but unnecessary—links that an agent might think worth maintaining.

Specifically, we consider a “flat” environment where *L*_*ij*_ = 0.95, i.e. that there is only a small efficiency loss, and there are no preferred (or excluded) agent-to-agent links. Since all links are (initially) allowed for use by any of the communications tactics, and links are equally efficient, all-averaged measures—e.g. averaged $\Phi_{b}^{a}$ or averaged functions thereof are useful as a test of performance.

**Fig 6:**
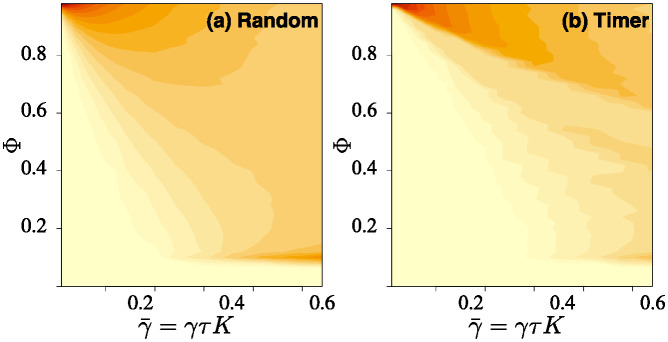
Filled contour plot based on histograms of the occurence-frequency of binned values of $\Phi_{j}^{i}$ and as a function of $\bar{\gamma }=\gamma \tau K$. Other parameters are *N* = 20, *K* = 200, *Φ*_m_ = 0.10, with all *L*_*ij*_ = 0.95. Each simulation, for the resulting agent-independent $\alpha_{j}^{i}=\alpha$, produces one histogram that makes up one vertical slice of the whole figure panel; thus we can scan the figure left-to-right and see how the behaviour will change as this loss ratio is increased. Here the color scale is the same on both panels, where dark stands for more likely, is based on the *cube root* of the occurrence-frequencies so as to improve feature visibility. On the left in (a), we see the results using the Random tactic (ii); and on the right in (b), the Timer tactic (iii). The results for the Sequential tactic (i) are very similar to those for Timer (iii).

Fig 6 shows a transition from a “good swarm” with much high-probability and accurate agent beliefs, and, as the normalised decay rate increases, changing to a fragmented (non) swarm with only the floor probability Φ_m_ remaining. There is a transition region where the swarm starts to fail, where the accuracy values of $\Phi_{b}^{a}$ are more widely distributed. Here we see that the Timer tactic performs better than the Random one, as the frequency of high-value $\Phi_{j}^{i}$ is better.

**Fig 7:**
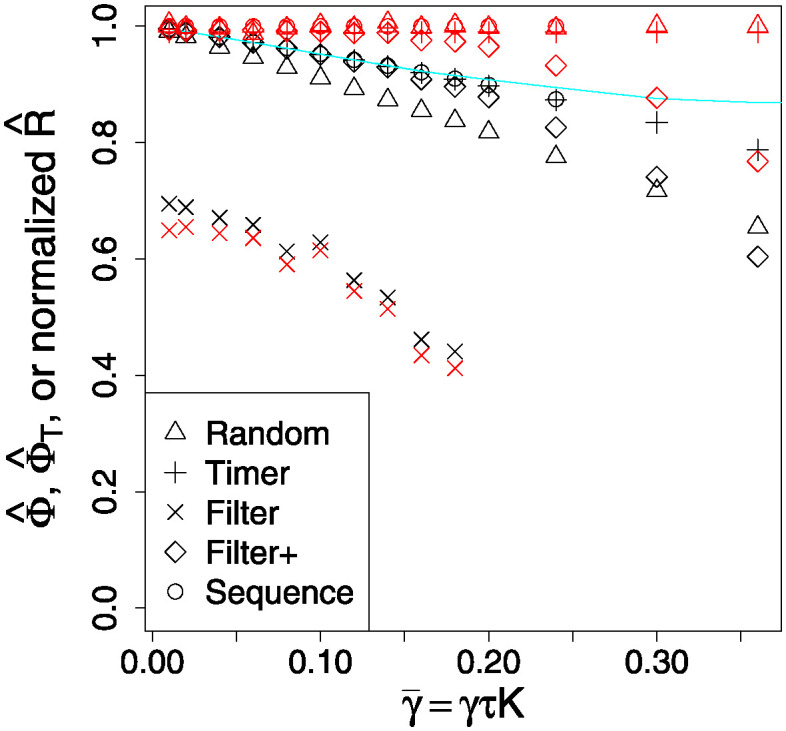
Comparison of outcomes in both performance $\hat{\Phi }\left(1\right)$ (black symbols) and normalised risk $\hat{R}$ (red symbols) for the different communications tactics; we want risk to be low, and performance to be high. The “missing” ×, ∘ points at high $\bar{\gamma }$ are omitted for cases where no CCS was formed. The cyan line is that for the ${\hat{\Phi }}_{\text{T}}$ measure, which in this plot is nearly the same for every tactic. Note that $\bar{\gamma }$ is the counterpart of *r* in the continuum model.

In Fig 7 we see that in terms of swarm performance, the Random communications tactic is typically the least advantageous, with poor connectivity $\hat{\Phi }$ and high risk $\hat{R}$. We should expect this, since some links will, by chance, and albeit temporarily, be neglected by an agent, leading to its accuracy dropping further. In contrast, the Timer and Sequence tactics are more regular in messaging over links, leading to a more compact distribution of accuracies (see Fig 6), and higher values (Fig 7). However, all three tactics have a similar risk profile because they are subject to the same rate limiting, and do not ignore any link possibilities.

The results for the Filtered tactic here are only indicative, and not strictly in a quasi-steady state. This is because this tactic chooses to prohibit sending on some links, but will never re-enable them, so that even good links will—in principle—eventually have a sufficiently unlucky period that results in them being switched off. An all-links switch-off can be seen at about 30k ticks into the simulation, but only at extremely high losses $\bar{\gamma }>0.45$, is still in-progress for $0.23<\bar{\gamma }<0.45$, but not yet evident for $\bar{\gamma }<0.23$. The Filtered+ and Filtered++ tactics, with their additional random transmission targets, significantly delay this process, but do not entirely stop it.

### 6.2 Partially connectable swarm

**Fig 8:**
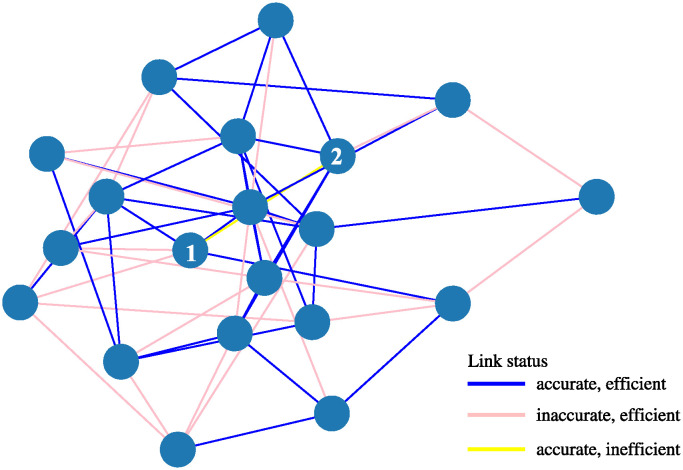
Background environmental network as defined by *L*_*ij*_, shown with link colours indicating the final simulation state of a simulation with a relatively large loss $\bar{\gamma }=0.24$, and using the Filter+ tactic. Blue lines are links that are both efficient (environmental) and that are known accurately, whereas the light pink ones are efficient but inaccurately known (i.e. “lost” links”); the partly obscured yellow line is an inefficient link between agents 1 and 2 that by luck has unexpectedly become accurate. Although each agent in this diagram is indeed linked to each other agent via intermediate agents, for some of those paths *more* than three hops are required, and so a CCS is not present here.

Next we consider a partially connectable swarm, i.e. we choose an environment where *L*_*ij*_ is randomly chosen to be either zero or one, forming a swarm of agents that are connected to only a few neighbours, but which are connected enough so that they can in principle still be globally connected. Here we generate the same random network for each different loss value tested, with a link probability of *p* = 0.284, and one instance of the resulting connectivity can be seen in Fig 8. The idea here was to ensure that the swarm was better connected than a “just connected” ER network with *p* = log(*N*)/*N*, which for *N* = 20 gives *p* ≃ 0.150, so that there are some redundant links, but not too many. This boost in link probability also helps improve connectivity, since at *N* = 20 we are not in the large *N* limit where the ER criteria is valid.

**Fig 9:**
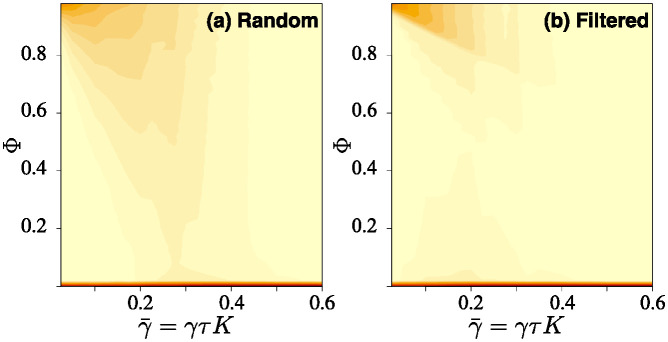
Filled contour plot based on histograms of the occurence-frequency of binned values of $\Phi_{j}^{i}$ and as a function of rate ratio $\bar{\gamma }=\gamma \tau K$. Other parameters are *N* = 20, *K* = 200, Φ_m_ = 0.00, with the *L*_*ij*_ being randomly 0 or 1 as described in the main text. Each simulation, for some $\bar{\gamma }$, produces one histogram that makes up one vertical slice of the whole figure panel; thus we can scan the figure left-to-right and see how the behaviour will change as loss is increased. Here the color scale is the same on both panels, where dark stands for more likely, is based on the *cube root* of the occurence-frequencies so as to improve feature visibility. On the left in (a), we see results using the Random tactic (ii), and on the right in (b), the Filtered tactic (iv). The results for Timer and Sequential tactics are very similar to Filtered, but with better high $\bar{\gamma }$ performance.

Fig 9 show transitions away from a “good swarm” with much high-probability and accurate agent beliefs, through to a fragmented (non) swarm with only the floor probability Φ_m_ remaining. Compared to Fig 6, the good swarm regime persists for a smaller range of information decay strengths, as might be expected given the much reduced number of efficient links.

**Fig 10:**
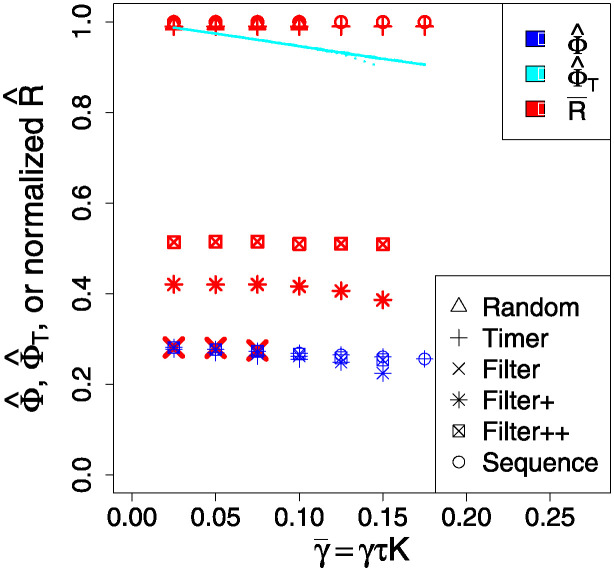
Comparison of outcomes in both performance $\hat{\Phi }\left(1\right)$ (blue symbols) and scaled risk $\hat{R}$ (red symbols) for the different communications tactics; we want risk to be low, and performance to be high. The “missing” points at higher $\bar{\gamma }=\gamma \tau K$ are omitted for cases where no CCS was formed. The cyan line(s) are those for the ${\hat{\Phi }}_{\text{T}}$ measure, which in this case are nearly the same for every tactic, and here are *the* most relevant metric for swarm performance.

Since only a fraction of the *L*_*ij*_ are non-zero, the simple performance measure $\hat{\Phi }$ is low, because it reflects this fraction—accuracies cannot be maintained if update messages never arrive. In contrast, the thresholded performance measure ${\hat{\Phi }}_{\text{T}}$ remains high as “good links” can easily persist, although in Fig 10 we can see this drop off for higher losses. A key feature showing differences here is the normalised risk measure $\bar{R}$. For the Random, Timer, and Sequence tactics it is the same (at ∼ 1), as expected since these tactics all transmit packets at the same average rate, regardless of efficiencies or accuracies. In contrast, the Filtered tactics exhibit much reduced risks, although the variants with extra “top up” updates are more risky, as would be expected.

On Fig 10 we see that the “all links” performance measure $\hat{\Phi }$ is uniformly low, being approximately the same as the link probability used to generate the *L*_*ij*_; which implies that efficient links– as might be expected—lead to agents having high accuracy Φ values on those links. This is confirmed by the thresholded performance measure ${\hat{\Phi }}_{\text{T}}$, which remains high, because it depends only on accurate belief values.

**Fig 11:**
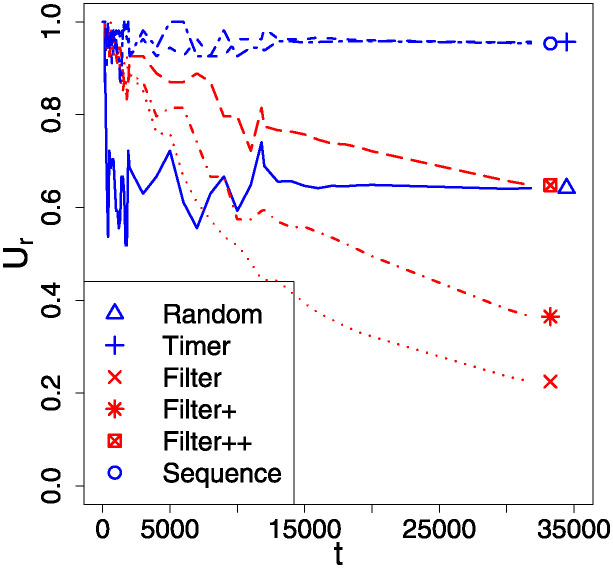
Swarm-averaged relative link counts *U*_*r*_ as a function of simulation tick-count *t* for the different transmission tactics. *U*_*r*_ is the number of found accurate links (as determined by $\Phi_{j}^{i}>\Phi_{\text{PT}}$), divided by the total number of efficient links (as determined from *L*_*ij*_ > Φ_PT_). We see that Random, Timer, and Sequential find steady state values, but as time passes the Filtered tactics, if not stopped, lose the use of more and more links. These results are for a relatively high loss of $r=\bar{\gamma }=\gamma \tau K=0.20$, selected to emphasise the different behaviors, but are much less extreme for smaller $\bar{\gamma }$ values. Note that $\bar{\gamma }$ is the counterpart of *r* in the continuum model.

It should be noted again that although a useful steady state limit for the Random, Timer, and Sequence tactics is possible, in the long term the Filtered tactics will all gradually lose even good links to bad luck, as seen on Fig 11. Thus on Fig 10, the Filtered-type results are only indicative of a medium term performance.

## 7 Summary

The preceeding results have shown fundamental scoping and initial results for our scenario considering agent-swarm management under stringent communications constraint. In particular, we have:

1. developed an agent-based information model allowing both a continuum and discrete communications paradigm; as well as a way of selecting inter-agent communications links that are preferential (more efficient) for use than others, thus mimicking the effect of a spatial distribution of agents without extra complications.
2. discussed the need for a distinction between the contents of the model (in particular $\Phi_{j}^{i}$, *γ*, *L*_*ij*_) and what quantities an agent *a* should be allowed to use when taking action (e.g. not any of *L*_*ij*_).
3. presented metrics related to communications performance (i.e. $\hat{\Phi }$ and ${\hat{\Phi }}_{\text{T}}$) and risk ($\hat{R}$) that can be used to evaluate both aspects of this performance-under-constraint scenario.
4. proposed a “round trip time” test that agents can use to deprecate transmitting updates over inefficient links with small *L*_*ij*_, and which relies only on explicitly agent-knowable timing data (i.e. the Filter, Filter+, and Filter++ communications tactics).

We showed that the Filter tactics did reduce the usage of inefficient links without significantly affecting performance, at least in the medium term, and that improvements (i.e. Filter+ and Filter++) can greatly moderate the long time failure of the unmodifed Filter tactic. Although not addressed in this initial study, which is only intended to introduce the problem, modifications to these tactics could not only stop the long term failure, but would e.g. enhance responsiveness to changes in the environment *L*_*ij*_. Nevertheless, this double-edged behaviour from the Filter tactics does act a salutary warning that such tactics need to be checked for possible failure modes as well as for their adaptability.

It is important to note that here we have attempted as far as possible to use performance measures that are calculated in ways that only depend on agent properties, and then preferably on properties that an agent is *aware* of, in combination with plausible threshold parameters. This is deliberate, because agents need metrics based only on what they are aware of; and since the (e.g.) *L*_*ij*_ are explicitly unknowns, they should not be used. Nevertheless, because our information model is so abstract, we can only judge whether an agent thinks the *a* ↔ *b* link is accurate by using $\Phi_{b}^{a}$ regardless. However, we can still assume the results of such shortcuts based on $\Phi_{b}^{a}$ values are somehow representative of a comparable judgement an agent might make based on an actual basket of data containing locations and uncertainties. Only in Fig 11 do we have recourse to non-agent properties, but that is to achieve a normalization based on thresholded *L*_*ij*_ values, to estimate to what extent the swarm might have accurate information about the efficient links that are present.

## 8 Future work

The simple outcomes presented here act as an introduction to the more sophisticated considerations which arise when the scenarios become more realistic. Notably, in ongoing work we have considered signal transmission based on additive gaussian white noise and bit error rate methods, and the concommittant effects on signal ranges, all of which allow more realistic computations of *L*_*ab*_; as well as the role of message lengths, of transmission angular spread, power dependence, and others [37].

In such an extended context, the nature of the scenario’s adversary model becomes particularly important. Our model here concerns itself only with risk of detection by an external listening adversary, which we see as the primary threat to any initially stealthy swarm. Nevertheless, in future work we aim to consider threat models in more detail. These will involve not only considerations about adversary locations and signal detectability [37], but also could extend to the possibility of an intruder masquerading as a legitimate peer.

Further, the specific information sent between agents needs to be considered—not only to what extent it might be abbreviated (to reduce message lengths) or encrypted, but how it might be used to assist in coordinating the swarm. Here we only proposed some baseline communications tactics designed to function without active intervention. Notably, a key weakness of simple adaptive approaches (such as our Filtered tactic) is the possibility of permanently avoiding links that were only transiently unreliable, or which only appeared unreliable as a result of bad luck. Nevertheless, the risk minimisation of these Filtered tactics, achieved by avoiding unreliable links, could still be retained. We plan to investigate a range of strategies, e.g. by simply stopping the removal of filtered links after some suitable multiple of *K* ticks, or setting a minimum number of links for any agent, to assist with this.

Another area of future interest is the role of performance measures. Here we have focussed on agent-centric metrics, since it is fundamental that each agent needs to be able to judge its performance for itself, i.e. without relying on sending or receiving additional messages. Nevertheless, in simulation is is possible to take an omniscient view and compute swarm-level performance measures directly from the simulation state, and these can provide a valuable benchmark against which we can compare agent-only judgements. Of course there are many type of possible performance measure, from the low level “how well is the swarm connected” ones relevant to this initial investigation, to much higher level ones such as adaptability to a changing environment, or even success at some assigned task.

## 9 Conclusion

Here we have considered an abstract implementation of agent-swarm behaviour in terms of a mathematical multi-agent model. We presented a continuum communications model which allowed some analytic treatment and mainly focussed on discrete communications. This stochastic implementation is designed to be applicable to any likely implementation. In particular, the stochastic version also provides us with information about outcome distributions as well as average- behaviour; something the continuum model does not generate.

We can see how these distributions are key to understanding the success/failure threshold of the swarm as seen in Figs 6 and 9. Whilst in the clear-success and clear-failure regimes (i.e. either low-loss or high-loss) the distribution of information available to the agents is indeed narrowly peaked, we see that in the transition region it broadens asymmetrically. This behaviour is key since we consider scenarios where we must operate our swarm on minimal communications, and *it is somewhere in this transition region* that we would aim to position our operating parameters—i.e. in a “stealth” scenario, with enough messaging to ensure swarm coherence, but only *just* enough, so that risk of detection remains at a minimum. One might easily imagine an agent switching communications tactics in order to return to this transition region after events have either reduced or increased its swarm knowledge too much.

Although the distributions in performance are key to a detailed understanding, we also used summary metrics, notably the swarm risk $\hat{R}$ and the thresholded performance measure ${\hat{\Phi }}_{\text{T}}$. By thresholding, we enable the metric to ignore intrinsically inefficient and unnecessary links, which should indeed be irrelevant to any practical performance measure. Further, as part of the thresholding we normalised on the basis of a minimal ER network structure, so that poorly linked agents were still penalised in the metric, but in a way not dependent on any information specific to a particular set of *L*_*ij*_. One improvement that could be made here is to replace this minimum ER criterion with one specific to spatially distributed agents, rather than probabilistically linked ones.

The model proposed and demonstrated here was abstract but is nevertheless adaptable to future realistic agent-swarm implementations across a number of different application areas where communications are limited. We intend that this new algorithmic framework will serve as a foundation for more practical mission scenarios and with greater and more sophisticated engineering detail to be added in further implementations of this work in the future [37].

## Supporting information

S1 Appendix The continuum model steady state. (PDF)

S1 File Data for Figs 6, 7 and 9–11. (ZIP)
