# Supplementary material for: Agent swarms: Cooperation and coordination under stringent communications constraint
Source: PLoS One. 2024 Dec 11;19(12):e0311513. doi: 10.1371/journal.pone.0311513 (PMC11633978; doi:10.1371/journal.pone.0311513)
Supplement: S1 Appendix — (PDF) [file pone.0311513.s001.pdf]

Agent swarms: cooperation and coordination under stringent communications constraint

Paul Kinsler (dr.paul.kinsler@physics.org),
Sean Holman, Andrew Elliott, Cathryn N. Mitchell, R. Eddie Wilson.

PLOS ONE (2024) and arXiv:2210.01163

Appendix: The continuum model steady state

We can normalise the dynamical equation by scaling with respect to γ, so that with $s = \gamma t$, we have a dynamical equation for any accuracy, which is

$$\frac{d}{ds}\Phi_b^a = -\left(\Phi_b^a - \Phi_\mathrm{m}\right) + \frac{\alpha_a^b L_{ba}}{\gamma}\Phi_a^b\left(1-\Phi_b^a\right) \tag{20}$$

$$= -\left(\Phi_b^a - \Phi_\mathrm{m}\right) + \frac{1}{R_{ab}}\Phi_a^b\left(1-\Phi_b^a\right), \tag{21}$$

where $R_{ab} = \gamma/\alpha_a^b L_{ba}$.

Thus the steady state un-normalised (& normalised) accuracy Φ_b^a is

$$\Phi_b^a = \frac{\gamma\Phi_\mathrm{m} + \alpha_a^b L_{ba}\Phi_a^b}{\gamma + \alpha_a^b L_{ba}\Phi_a^b} \quad = \frac{\Phi_\mathrm{m} + R_{ab}^{-1}\Phi_a^b}{1 + R_{ab}^{-1}\Phi_a^b}, \tag{22}$$

and we can substitute either of these expressions into itself (with with reversed a, b indices), to get a polynomial for Φ_b^a.

We create a forward loss rate $r = \gamma/\alpha_a^b L_{ba}$ and a backward rate $r' = \gamma/\alpha_b^a L_{ab}$, so that we have

$$\Phi_b^a = \frac{r\Phi_\mathrm{m} + \Phi_a^b}{r + \Phi_a^b} \tag{23}$$

$$= \frac{r\Phi_\mathrm{m} + \frac{r'\Phi_\mathrm{m} + \Phi_b^a}{r' + \Phi_b^a}}{r + \frac{r'\Phi_\mathrm{m} + \Phi_b^a}{r' + \Phi_b^a}} \tag{24}$$

$$\Phi_b^a\left\{r\left[r' + \Phi_b^a\right] + \left[r'\Phi_\mathrm{m} + \Phi_b^a\right]\right\} = r\Phi_\mathrm{m}\left[r' + \Phi_b^a\right] + \left[r'\Phi_\mathrm{m} + \Phi_b^a\right] \tag{25}$$

$$r'\left[r + \Phi_\mathrm{m}\right]\Phi_b^a + \left[r+1\right]\left[\Phi_b^a\right]^2 = r'\Phi_\mathrm{m}\left[r+1\right] + \left[r\Phi_\mathrm{m} + 1\right]\Phi_b^a \tag{26}$$

$$\left[r'r + \left(r'-r\right)\Phi_\mathrm{m}\right]\Phi_b^a + \left[r+1\right]\left[\Phi_b^a\right]^2 = r'\Phi_\mathrm{m}\left[r+1\right] + \Phi_b^a. \tag{27}$$

Hence

$$\left[r+1\right]\left[\Phi_b^a\right]^2 + \left[r'r + \left(r'-r\right)\Phi_\mathrm{m} - 1\right]\Phi_b^a - r'\Phi_\mathrm{m}\left[r+1\right] = 0 \tag{28}$$

And if $r = r'$, which would be reasonable for a symmetric environment $L_{ab} = L_{ba}$, where both agent a and b were transmitting at the same default rate, we have the simpler form

$$\left[\Phi_b^a\right]^2 + \left[r-1\right]\Phi_b^a - r\Phi_\mathrm{m} = 0, \tag{29}$$

an expression which has just two free parameters, the rate ratio r and the minimum information Φ_m.

Thus

$$\Phi_b^a = \frac{1-r}{2} \pm \frac{1}{2}\sqrt{r^2 - 2r + 1 - 4(-r).\Phi_{\mathrm{m}}}, \tag{30}$$

$$= \frac{1}{2} - \frac{r}{2} \pm \frac{1}{2}\sqrt{r^2 - 2r\left(1 - 2\Phi_{\mathrm{m}}\right) + 1}. \tag{31}$$

If $\Phi_{\mathrm{m}} = 0$, then

$$\Phi_b^a = \frac{1}{2} - \frac{r}{2} \pm \frac{1}{2}\left(r-1\right), \tag{32}$$

which has two solutions; firstly the zero accuracy case $\Phi_b^a = 0$, and secondly the finite-accuracy case $\Phi_b^a = 1 - r$. However, if $\Phi_{\mathrm{m}} > 0$, the “zero accuracy” solution (sign choice “+”) is pushed negative so that only the finite-accuracy one remains.
